# Achieving competitive employment for individuals with intellectual disabilities in Saudi Arabia: a systematic review of barriers and facilitators

**DOI:** 10.3389/fpsyg.2026.1850048

**Published:** 2026-08-06

**Authors:** Sarah Khaled Alfawzan

**Affiliations:** Department of Special Education, College of Education, King Faisal University, Al-Ahsa, Saudi Arabia

**Keywords:** barriers, employer, employment, facilitators, intellectual disability, sustainability

## Abstract

**Background:**

Employment holds substantial meaning and provides important benefits for individuals with intellectual disabilities. However, they often encounter greater challenges in securing and maintaining employment compared to their non-disabled peers.

**Objective:**

To enhance understanding of the barriers and facilitators influencing the employment and job retention of individuals with intellectual disabilities from the perspectives of stakeholders in Saudi Arabia.

**Method:**

Web of Science, Scopus, ProQuest, and ERIC-EBSCO Mandumah databases were systematically searched to identify peer-reviewed studies on the employment of individuals with intellectual disabilities published between 2011 and 2025.

**Results:**

The findings highlight employer-related barriers, including discrimination and misconceptions, as well as challenges associated with education and vocational training and the skill development of individuals with intellectual disabilities. The results also identified key facilitators, including collaborative partnerships, accessibility services, and supportive workplace relationships. The study underscores the need to redesign secondary school curricula to strengthen vocational training and transition services, thereby enhancing the professional skills required in the labor market. It also emphasizes the importance of promoting a culture of inclusion and diversity in the workplace through targeted employer training and the implementation of accountability mechanisms within employing institutions.

## 1 Introduction

The number of individuals with disabilities worldwide is approximately 1.3 billion, with 200 million facing challenges in the workplace (World Health Organization, 2023). Disability and unemployment have received global attention due to their importance in achieving the Sustainable Development Goals, including poverty eradication (Saunders and Nedelec, 2014), and promoting the social inclusion of individuals with intellectual disabilities through integrated employment, interaction with non-disabled colleagues, and equal working conditions (Smith et al., 2019). Employment represents a form of community participation that extends beyond economic sustainability, enabling individuals to engage in productive activities, socialize, and achieve independence (Hästbacka et al., 2016). Participation in the workforce offers significant benefits for individuals with intellectual disabilities. Those employed in inclusive settings often experience improved quality of life, including higher standards of living, greater sense of belonging, enhanced wellbeing and self-esteem, and improved independence and communication skills (Robertson et al., 2019; Voermans et al., 2021). Despite international efforts, such as the United Nations Strategy for the Inclusion of Persons with Disabilities, which emphasizes removing institutional barriers, promoting inclusive employment practices, and improving equitable access to jobs (United Nations, 2019), and Article 27 of the Convention on the Rights of Persons with Disabilities, which requires States Parties to ensure access to inclusive labor markets and reasonable accommodations (United Nations, 2006), individuals with disabilities continue to face persistent employment barriers.

Recent data highlight this disparity. The 2024 US and UK Annual Disability Statistics Collection reported that only 39% of individuals with cognitive disabilities aged 18–64 were employed, compared to 77.2% of individuals without disabilities (Thomas et al., 2024). This gap contributes to lower employment and income levels among individuals with disabilities (Konrad et al., 2013; Thomas et al., 2024), with many remaining unemployed (Cimera et al., 2014). Exclusion from employment persists across multiple countries, including Australia, the United States, the United Kingdom (Butterworth et al., 2015; Riches and MacDonald, 2016), and Norway (Wendelborg et al., 2022). Similar challenges are evident in Saudi Arabia, where individuals with intellectual disabilities continue to encounter barriers to employment (AlDosari, 2016; Almalky, 2018).

### 1.1 Employment of individuals with disabilities in Saudi Arabia

Numerous regulations and laws in Saudi Arabia affirm the rights of individuals with disabilities. Article 2 of the Law on the Care of Individuals with Disabilities emphasizes their rights across several domains, including employment. It affirms their right to work in jobs suited to their abilities and qualifications, enabling them to realize their potential and earn income comparable to other members of society, while also promoting skill development through training (Disabled Care System, 2000). Furthermore, Article 10 of the Law on the Rights of Individuals with Disabilities guarantees the right to employment without discrimination. It mandates the development and implementation of employment programs, including vocational and technical training, and encourages both governmental and non-governmental employers to recruit individuals with disabilities. It also requires the adaptation of work systems and environments to meet their needs and ensure equal employment opportunities (System for Rights of Persons with Disabilities, 2023). In addition, Article 28 of the Saudi Labor Law requires employers with 25 or more employees to ensure that at least 4% of their workforce consists of individuals with disabilities (Board of Experts at the Council of Ministers, 2005). The law further obligates employers to create inclusive and supportive work environments and to provide equal opportunities in hiring, promotion, and retention without discrimination based on disability (System for Rights of Persons with Disabilities, 2023).

In response to recent changes in the Saudi labor market, the government has enacted several initiatives to support the employment and empowerment of individuals with disabilities. For example, the Nitaqat program, aligned with Saudi Vision 2030, aims to provide decent employment opportunities, create safe and attractive work environments, and reduce unemployment rates. The program offers incentives to companies that employ individuals with disabilities, counting one employee with a disability as four employees for Saudization targets (Ministry of Human Resources and Social Development, 2019). In addition, the Tawafuq program encourages the employment of individuals with disabilities in the private sector by facilitating access to suitable job opportunities aligned with their abilities and qualifications. The program also emphasizes the provision of supportive services to enable individuals to perform their job duties effectively and ensures that work environments are accessible and accommodating (Social Resources Development Fund, 2020). Furthermore, the Saudi government provides financial support to individuals with disabilities to cover costs related to job searching, training, and transportation. It also offers incentives to employers to support training and employment opportunities for individuals with disabilities (Ministry of Human Resources and Social Development, n.d.).

### 1.2 Employing individuals with intellectual disabilities in Saudi Arabia

The development of laws and legislation supporting the employment and empowerment of individuals with disabilities has contributed to progress in rehabilitation, training, and employment programs, as well as initiatives aimed at facilitating their integration into the labor market (Almalky and Alqahtani, 2022). These efforts include creating supportive and accessible work environments that address not only physical accessibility but also job selection procedures, workplace adaptations, flexible working hours, and the provision of assistive technologies, all intended to reduce discrimination, improve access to employment, and address negative perceptions of productivity (Alsharadqah and AlGhamdi, 2024). Despite these efforts, relatively few individuals with intellectual disabilities successfully transition into the workforce (AlDosari, 2016; Almalky, 2018). In Saudi Arabia, individuals with disabilities represent approximately 1.8% of the total population, and 9.3% of unemployed individuals have never worked (General Authority for Statistics, 2023). Employment rates among individuals with disabilities remain low compared to the general population, and many require additional support to participate in the workforce (Sundermann et al., 2023), resulting in a substantial proportion remaining at home after completing school.

### 1.3 Challenges in employing individuals with intellectual disabilities in Saudi Arabia

Individuals with disabilities in Saudi Arabia face multiple employment challenges, including limited access to training and job opportunities and a lack of understanding of the disability population (Almalky, 2018). In addition, inaccurate perceptions of individuals with intellectual disabilities among stakeholders contribute to discrimination and bias in employment (Abed et al., 2024; Amreen and Ghareeb, 2025). There is also a gap in the role of education in developing vocational skills essential for workplace success and in addressing negative stereotypes (Almughira, 2024; Alzarie, 2024). These challenges highlight the need to identify and address barriers, improve employment outcomes, and expand research to bridge this gap and strengthen the existing body of literature.

### 1.4 Aims

This systematic review aims to (a) identify barriers to employment for individuals with intellectual disabilities in Saudi Arabia and (b) propose facilitators to address them. The review responds to the need to examine employment conditions for individuals with intellectual disabilities to inform the development of targeted interventions, employment programs, and support initiatives. These efforts are expected to enhance participation and contribute positively to Saudi society. Furthermore, the review offers recommendations to stakeholders to support the employment of individuals with intellectual disabilities.

## 2 Materials and methods

### 2.1 Search strategy

On 7 March 2025, five databases were searched: ERIC–EBSCO, Mandumah, ERIC–ProQuest, Web of Science, and Scopus, using a search string that included terms related to the employment of individuals with intellectual disabilities (see Table 1). The search was limited to studies published in Arabic or English. The primary search string used in one of the databases was: (“Employment” OR “Supported Employment” OR “Inclusive Employment” OR “Integrated Employment” OR “Open Work” OR “Employer”) AND (“Barriers” OR “Challenges” OR “Obstacles” OR “Solutions” OR “Positive Influencing Factors” OR “Enabling Factors” OR “Facilitators”) AND (“Kingdom of Saudi Arabia” OR “SA” OR “KSA”) AND (“Intellectual Disability” OR “Mental Retardation” OR “Down Syndrome” OR “ID”) AND (“Qualitative” OR “Quantitative” OR “Mixed Approach”) AND (“2011”–“2025”). In addition, the reference lists of included articles were manually reviewed to identify further studies.

**Table 1:** Search terms EBSCO, Mandumah, ERIC–ProQuest, Web of Science, Scopus.

Concept	Search words
Topic	Employment, employer, integrated/inclusive employment, supported employment, open work, barriers, challenges, hurdles, solutions, positive influencing factors, enablers, facilitators
Country	Saudi Arabia, SA, KSA, Kingdome Of Saudi Arabia
Target population	Mental retardation, intellectual disability, down syndrome, ID
Methodology	Qualitative, quantitative, mixed-method designs
Publication dates	Between 2011 and 2025
Language	English and Arabic

### 2.2 Eligibility criteria

To complete the article selection, several inclusion criteria were applied to the titles, abstracts, and full texts: (1) studies employed qualitative, quantitative, or mixed-methods designs; (2) studies focused on individuals with intellectual disabilities; (3) studies addressed barriers and facilitators to employment in Saudi Arabia and explored stakeholder perspectives, including teachers and transition team members, human resources managers, parents, employers and supervisors, individuals with intellectual disabilities, and their trainers; (4) studies were published in Arabic or English; and (5) studies were published in peer-reviewed journals between 2011 and 2025, with full text available through open access or institutional subscriptions to enable comprehensive evaluation. Ali (2011) highlighted the importance of employment as a core value for individuals with intellectual disabilities and emphasized the need to examine stakeholder perspectives on their employment. In response to subsequent political and societal shifts in Saudi Arabia supporting the employment of individuals with intellectual disabilities, this timeframe was selected to capture developments from 2011 to the present. Although a global review examined barriers to employment for individuals with intellectual disabilities (AlFozan and AlKahtani, 2021), no systematic review specific to Saudi Arabia focusing on this population was identified. That review synthesized international evidence but did not address the unique policy and labor market developments the Kingdom has experienced over the past 5 years. Given the substantial expansion of national initiatives supporting individuals with disabilities and the increasing number of studies incorporating the perspectives of individuals with disabilities, a contemporary review tailored to the Saudi context is essential to capture these developments, examine recent employment research involving individuals with intellectual disabilities, and contribute to understanding the barriers and facilitators to employment while enriching knowledge in this field.

### 2.3 Exclusion criteria

The following studies were excluded: (1) studies that did not include participants with intellectual disabilities or addressed broader disability categories without reporting specific findings for this group; (2) studies that did not focus on Saudi Arabia; (3) studies that addressed issues unrelated to the employment of individuals with intellectual disabilities; (4) literature reviews, opinion articles, and editorials; (5) articles not published in peer-reviewed journals or conference proceedings; (6) studies that did not clearly describe data collection methods or lacked participant demographic information; and (7) articles not written in English or Arabic, published outside the 2011–2025 period, or without accessible full text.

### 2.4 Selection and procedures for studies

According to Preferred Reporting Items for Systematic Reviews and Meta-Analyses (PRISMA) guidelines (Liberati et al., 2009), the selection process consists of four phases: identification, screening, eligibility, and inclusion. The initial search yielded 11,658 articles. An iterative search strategy was employed, involving continuous refinement of search terms and criteria to progressively filter results, exclude irrelevant studies, and retrieve literature most relevant to the research questions. After all records were imported into Zotero reference management software, the number was reduced to 565 articles following the removal of duplicates and clearly irrelevant records. These exclusions were based on technical criteria rather than full-text evaluation. Specifically, excluded records included documents that did not meet the required study type (e.g., books or ineligible articles), results generated by partial or inaccurate keyword matches (e.g., studies using terms such as “employment” or “intellectual” in unrelated contexts), and articles outside the study's scope, such as medical or economic research that did not address employment or intellectual disability despite containing similar keywords.

The initial screening, based on titles and abstracts, identified 241 articles for further evaluation. Of these, 31 articles could not be accessed because the full text was unavailable, and 191 articles were excluded because they did not focus on employment in Saudi Arabia, included populations other than individuals with intellectual disabilities, were published in non-peer-reviewed sources, or were classified as systematic reviews or meta-analyses, or because they were not published in Arabic or English. This exclusion followed the researcher's independent review of each record using a standardized checklist aligned with the predefined inclusion and utilization criteria. During the full-text screening phase, the researcher examined all eligible articles in detail, followed by a second verification round to ensure consistency in the decision-making process. A total of 19 studies were included in the final review, representing a range of stakeholders and methodological designs relevant to the employment of individuals with intellectual disabilities. Data extraction focused on findings related to employment barriers and facilitating factors. The extraction framework was based on predefined variables in the review protocol, including author, year of publication, sample, country, research methodology, instruments used, and key findings. This framework was applied consistently across all included studies to ensure accuracy and comparability. The extracted information is summarized in Table 2.

**Table 2:** Sample, methodology, instruments, and results of the studies reviewed.

No.	References	Sample	Methodology	Instruments	Summary of results
1	Alzahrani (2025)	Seven trainers for individuals with intellectual and developmental disabilities	Qualitative	Semi-structured interviews	The findings emphasized the critical role of vocational training, parental support, education, and psychosocial wellbeing as key determinants of competitive employment for individuals with intellectual and developmental disabilities. The results further underscored the importance of supportive and well-adapted work environments, along with adequate resources and assistance, to enable these individuals to achieve their full potential
2	Amreen and Ghareeb (2025)	Six employers	Qualitative	Semi-structured interviews	The findings identified three categories of challenges that hinder the employment of individuals with intellectual disabilities: (1) employer-related challenges, (2) challenges related to the individual, and (3) challenges associated with responsible authorities. The study also proposed several strategies to enhance employment opportunities, including: (1) establishing a specialized government agency to provide training and facilitate job placement, (2) strengthening collaborative partnerships among relevant stakeholders, and (3) utilizing modern media to promote awareness and support for their employment
3	Abed et al. (2024)	Five employers and five individuals with disabilities	Qualitative	Semi-structured interviews	The findings identified several challenges, including misconceptions, ineffective legislation, limited training and educational opportunities, and factors related to the type, age, and gender of disability. The results also highlighted key strategies to address these barriers, including increasing awareness, implementing inclusive policies, providing reasonable workplace accommodations for individuals with disabilities, and ensuring access to appropriate technologies to support task performance
4	Alzarie (2024)	Six employees with mild intellectual disabilities	Qualitative	Interviews	The findings indicate that problem-solving skills are a critical requirement in the workplace for individuals with mild intellectual disabilities. The study also highlights the significant role of self-determination in supporting employment success, emphasizing that this skill can be strengthened through practice and targeted training designed to enhance performance in work environments
5	Alsharadqah and AlGhamdi (2024)	142 Parents of Individuals with intellectual disabilities.	Quantitative	Questionnaire	The study findings indicated that the current state of rehabilitation and employment for individuals with intellectual disabilities was at a moderate level, while the obstacles to their rehabilitation and employment were reported to be high. The results also revealed statistically significant differences based on the level of disability. These differences favored individuals with Fragile X syndrome in terms of rehabilitation and employment outcomes, whereas individuals with Down syndrome experienced higher levels of barriers to rehabilitation and employment
6	Almughira (2024)	69 Employees of an institution for the rehabilitation and employment of persons with disabilities	Quantitative	Questionnaire	The study identified three categories of barriers to employment for individuals with intellectual disabilities. First, individual-related barriers included limited skills, insufficient training and qualification opportunities, and inadequate preparation for work. Second, family-related barriers involved concerns about social stigma, including fear of negative perceptions and the belief that employment opportunities may be based on pity rather than merit. Third, society-related barriers included limited access to training opportunities, narrow perceptions of their work capabilities, and inequities in employment rights, such as unequal access to training, promotions, and fair wages compared to their peers
7	Algharbi and Alsayed (2024)	83 Female teachers of intellectual education in secondary school	Quantitative	Questionnaire	The study findings indicated that assistive technologies play a significant role in enabling female students with intellectual disabilities to acquire vocational skills aligned with their abilities and to prepare for employment. The results also identified the lack of accredited vocational rehabilitation programs incorporating assistive technologies as a major barrier. Furthermore, the study emphasized the importance of integrating assistive technologies and modern digital applications within inclusive secondary school settings to enhance skill development and employment readiness
8	Alasimi and Aljahni (2023)	Six participants (three individuals with mild intellectual disabilities, all of whom are employed, and three parents)	Qualitative	Semi-structured interviews	The study findings indicated that secondary school plays a limited role in preparing individuals with intellectual disabilities for employment. The stigma associated with the term “intellectual disability,” which is often misunderstood or unrecognized by employers, further limits access to appropriate job opportunities. In addition, there is a lack of post-secondary and on-the-job training programs to support the development of essential work-related skills The results also highlighted a strong desire among participants for independence and the opportunity to establish families, which served as key motivations for seeking employment. Engagement in work contributed to a sense of accomplishment and self-reliance. However, participants reported the absence of employment incentives, such as health insurance and housing allowances, which may affect both job attraction and retention
9	Alsalmi and Balbaid (2023)	82 Parents of individuals with intellectual disabilities	Quantitative	Questionnaire	The results indicated that employment barriers for individuals with intellectual disabilities were substantial, with societal and environmental barriers ranked highest, followed by personal barriers, and family-related barriers in third place. Based on these findings, the study recommends increasing awareness among all members of society regarding the employment rights of individuals with intellectual disabilities and the positive impact of their inclusion across multiple aspects of life
10	Kaabi and Alqudah (2022)	118 Members of the staff in secondary intellectual education institutes and secondary schools	Quantitative	Questionnaire	The study findings indicated a moderate level of supported employment overall. No statistically significant differences were found based on gender across most dimensions, except for the “challenges” dimension, where results favored female participants. In addition, statistically significant differences were identified based on the type of educational institution, with results favoring these institutions across all dimensions except the “challenges” dimension
11	Elahdi and Alnahdi (2022)	168 Supervisors/owners, workers, and treasurers	Quantitative	Questionnaire	The study findings indicated that familiarity and prior work experience with individuals with intellectual disabilities were positively associated with employees' attitudes toward hiring them. In contrast, owners and supervisors demonstrated greater hesitation compared to employees, suggesting a lower level of professional support and confidence in employing individuals with intellectual disabilities within workplace settings
12	Alanazi (2021)	10 Individuals with intellectual disabilities	Qualitative	In-depth interviews	The results indicate that participants face multiple employment challenges, including mismatches with required skill levels, low employer confidence, perceptions of inability to meet job demands, assumptions of limited competence, and the provision of low wages
13	Almalki et al. (2022)	Four individuals with intellectual disabilities in Riyadh	Qualitative	Semi-structured interviews	The findings highlighted several opportunities associated with the initiative, particularly the strong moral support and positive attitudes of families toward individuals with intellectual disabilities. However, the results also identified key challenges, most notably the absence of laws mandating vocational training during secondary school years. Participants emphasized the importance of strengthening collaboration between schools and training institutions to ensure adequate preparation for successful entry into the labor market
14	Baothman (2021)	151 Teachers from institutes and programs for intellectual education at the secondary level, and all supervisors working in sectors employing Individuals with intellectual disabilities	Mixed	Questionnaire Semi-structured interview	The quantitative findings indicated that participants placed high importance on self-care skills, safety and security, work-related competencies, and communication and social interaction skills within the workplace. In contrast, the qualitative findings identified key themes, including the importance of career planning and the development of partnerships with sectors responsible for job placement and professional development
15	AlQahtani and AlDaaj (2020)	322 Parents of students with intellectual disabilities in secondary school	Quantitative	Questionnaire	The results indicated that the most significant barriers to the employment empowerment of individuals with intellectual disabilities were societal factors, followed by family-related barriers, then those related to the individuals themselves, and finally those associated with colleagues. The findings also identified key strategies to enhance employment empowerment, including establishing a dedicated entity to support the employment of individuals with disabilities, creating a regulatory body to oversee and monitor employment processes and ensure accountability, and providing financial and informational support regarding appropriate workplace accommodations
16	AlDosari and Muajini (2019)	388 Human resources managers in private sector companies	Quantitative	Questionnaire	The results indicated that individuals with intellectual disabilities often lack essential work-related skills and are not adequately prepared for employment. The most significant societal barriers included the mismatch between job requirements and their abilities, as well as the absence of safe and supportive work environments. Family-related barriers involved parental perceptions that employment is driven by pity rather than recognition of capability, in addition to concerns about the low wages offered to these individuals
17	AlDosari (2016)	43 Individuals who work directly with individuals with mild intellectual disabilities	Quantitative	Questionnaire	The study findings indicated that the obstacles affecting the success of individuals with mild intellectual disabilities in the workplace were substantial, as perceived by their colleagues. Statistically significant differences were observed in barriers related to social skills and transportation, legislation and regulatory frameworks, and academic skills, with higher levels of these challenges reported in private sector work environments
18	Alajami and Albatal (2016)	945 Employees in intellectual education institutes and their programs	Quantitative	Questionnaire	The study concluded that individuals with intellectual disabilities face multiple barriers to employment, including limited work-related skills, insufficient access to training, and difficulties in using technology. Additional challenges include a preference for foreign labor, limited availability of training opportunities, and ineffective parenting approaches. Family-related factors also play a role, including concerns about potential mistreatment by others and negative perceptions regarding the competence and abilities of individuals with intellectual disabilities
19	Ali (2011)	37 Families of individuals with disabilities, and 37 business owners	Quantitative	Interview-scale	The findings identified several family-related barriers affecting the employment of individuals with disabilities, including concerns about their safety, challenges related to transportation to and from the workplace, and experiences of harassment. In addition, employer-related barriers were reported, such as practices that hinder or restrict employment opportunities for individuals with disabilities

**Figure 1:**
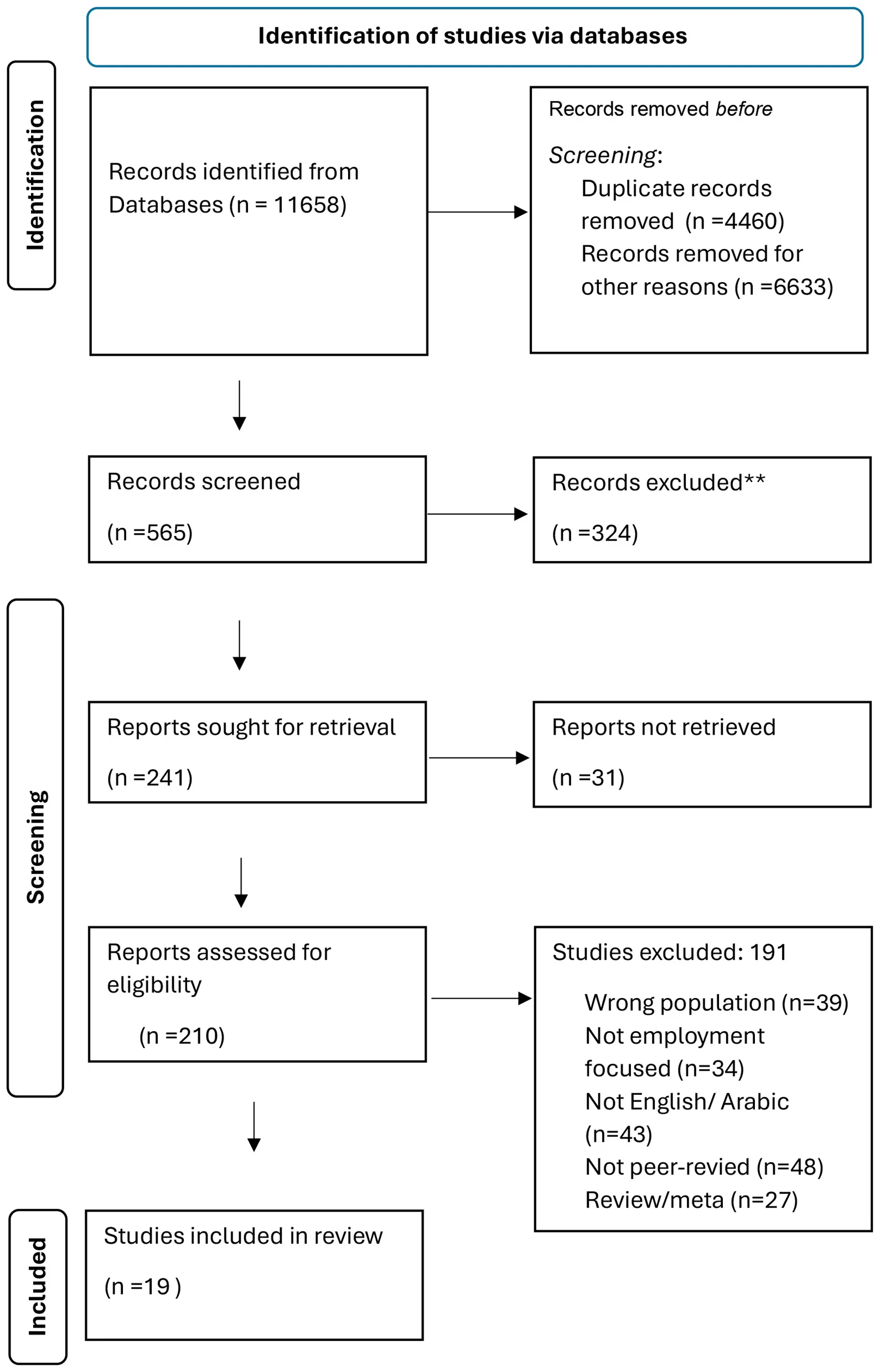
PRISMA flowchart of study selection.

Each study was reviewed repeatedly and summarized to ensure a thorough examination of the relevant literature. The extracted data were carefully assessed for relevance and validity. To maintain consistency and minimize potential bias, clear methodological procedures were followed, including two rounds of screening at each stage to ensure internal consistency, strict adherence to predefined inclusion and exclusion criteria, and the use of a standardized data extraction model for all included studies. The Mixed Methods Assessment Tool (MMAT) was also used to assess the quality of the included studies because of its suitability for evaluating quantitative, qualitative, and mixed-methods research. The assessment examined the appropriateness of the measures, sampling methods, and potential bias (Hong et al., 2018). The MMAT assessment showed that most studies included in this review were of high methodological quality. All seven qualitative studies (100%) met all five MMAT criteria and were classified as high quality. Of the 11 quantitative studies, eight (72.7%) were classified as high quality, whereas three (27.3%) were rated as average quality because of unclear information regarding sample representation or non-response bias. The single mixed-methods study met most criteria but showed some limitations in integrating qualitative and quantitative components. Overall, approximately 83% of the included studies were classified as high quality. These findings indicate a strong level of methodological rigor across most studies, strengthening the reliability of the evidence presented in this review. High-quality studies were given greater weight in drawing conclusions, whereas the methodological limitations of lower-quality studies were explicitly considered when interpreting the findings. The extracted data were then reviewed and coded line by line using word processing software. An inductive approach was used to code the findings and determine their relevance to the research question. Categories were subsequently identified, and codes with similar content and meaning were grouped systematically. Common barriers and facilitators were then identified. A summary of the selected studies, following the PRISMA guidelines, is presented below in Figure 1.

## 3 Results

### 3.1 What are the barriers to employment for individuals with intellectual disabilities in Saudi Arabia from the perspective of stakeholders?

Table 2 summarizes the barriers and facilitators to employment and retention for individuals with intellectual disabilities from the perspective of stakeholders in Saudi Arabia. Nineteen studies met the inclusion criteria: 11 quantitative, 7 qualitative, and 1 mixed-methods, employing interviews, focus groups, questionnaires, and scales. This methodological diversity provided a comprehensive understanding of employment barriers and facilitators from multiple stakeholder perspectives, including teachers and transition team members, human resources managers, parents, employers and supervisors, and individuals with intellectual disabilities, along with their trainers and support staff. The identified barriers were categorized into three groups: employer-related barriers, barriers related to education and vocational training, and barriers related to the individual with intellectual disabilities.

#### 3.1.1 Employer-related barriers

Studies have shown that employers present multiple challenges to the employment of individuals with intellectual disabilities (Ali, 2011), including discrimination and bias in hiring practices (Abed et al., 2024). Employers are often more hesitant to hire individuals with intellectual disabilities than non-disabled employees, partly due to stigma associated with the label “intellectual disability,” which can limit access to suitable employment opportunities (Alasimi and Aljahni, 2023). In addition, economic considerations and profit-driven priorities influence employer decisions, with concerns about potential financial losses and productivity often cited as reasons for reluctance to hire (Elahdi and Alnahdi, 2022). The findings also indicate that individuals with intellectual disabilities frequently do not receive equitable employment rights, including fair training opportunities and salaries comparable to their colleagues (Alanazi, 2021; Almughira, 2024). They are often excluded from promotions, bonuses, and incentives within their organizations (AlDosari, 2016; Alsalmi and Balbaid, 2023). Furthermore, employers may prefer foreign labor, perceived as lower cost and more productive, over individuals with intellectual disabilities (Alajami and Albatal, 2016). Employers and supervisors may also favor non-disabled employees due to perceptions that individuals with intellectual disabilities lack necessary communication and social skills for the workplace (Elahdi and Alnahdi, 2022).

Furthermore, the findings indicate that employers often hold misconceptions and inaccurate perceptions of individuals with intellectual disabilities, contributing to discrimination and bias in hiring (Abed et al., 2024). Even when employed, employers may lack confidence in their abilities, perceiving them as incapable, uncommitted, or prone to lateness and absenteeism (Abed et al., 2024). Employers may also view employing individuals with intellectual disabilities as costly, requiring continuous supervision and raising concerns about accountability (Abed et al., 2024; Amreen and Ghareeb, 2025). Additionally, employees with intellectual disabilities are frequently marginalized and not assigned meaningful tasks or responsibilities (AlDosari, 2016). Instead, tasks may be poorly matched to their abilities, often due to limited awareness and insufficient collaboration between employers and organizations that support the employment of individuals with intellectual disabilities (Amreen and Ghareeb, 2025).

#### 3.1.2 Obstacles related to education and vocational training

Stakeholders' perspectives indicate that some individuals with intellectual disabilities do not receive adequate education and training opportunities at the school level (Abed et al., 2024; Alasimi and Aljahni, 2023; Almalki et al., 2022). This limits their ability to develop functional skills and competencies (Abed et al., 2024). In addition, limited exposure to the labor market during schooling, including the absence of field visits and restricted training opportunities, sometimes limited to 1 day per week, further constrains skill development (Elahdi and Alnahdi, 2022). Weak transition services, potentially linked to the absence of policies mandating training during secondary education, also contribute to these challenges (Almalki et al., 2022). As a result, employers may be reluctant to hire individuals with intellectual disabilities due to inadequate preparation for the workforce, as services up to age 18 often emphasize academic content with limited non-academic training (Elahdi and Alnahdi, 2022). Consequently, secondary education may play a limited role in preparing individuals for employment (Alasimi and Aljahni, 2023).

These conditions contribute to limited training and experience among individuals with intellectual disabilities (Amreen and Ghareeb, 2025), resulting in insufficient work-related skills and reduced employment opportunities. This is associated with inadequate training, rehabilitation, and job preparation programs, as well as limited access to appropriate qualifications for effective job performance (Alajami and Albatal, 2016; AlDosari and Muajini, 2019; Almughira, 2024; Alasimi and Aljahni, 2023). Additional challenges include the limited availability of vocational training and rehabilitation centers, particularly for individuals with mild intellectual disabilities, and the limited alignment of existing vocational programs with labor market demands. Furthermore, vocational training programs often lack the practical components necessary to develop professional competencies and sustain long-term employment (AlDosari, 2016).

#### 3.1.3 Obstacles related to individuals with intellectual disability

The findings identified several challenges related to individuals with intellectual disabilities, including limited participation in job fairs, recruitment platforms, and workshops, which reduces their visibility to employers and may limit hiring opportunities (AlQahtani and AlDaaj, 2020; Amreen and Ghareeb, 2025). In addition, individuals may experience difficulty identifying suitable job opportunities, managing work demands, and completing assigned tasks effectively (Alanazi, 2021; AlQahtani and AlDaaj, 2020). Health-related factors may also affect job retention and reduce employment opportunities (Almughira, 2024). Some workplace challenges include difficulties adhering to expected professional behaviors, such as respecting colleagues' property and following instructions consistently (AlDosari, 2016).

The findings also indicate that individuals with intellectual disabilities may lack essential work-related skills (Alajami and Albatal, 2016; AlDosari and Muajini, 2019; Alsharadqah and AlGhamdi, 2024), including communication difficulties (Amreen and Ghareeb, 2025), limited attention and concentration, and challenges expressing needs (Alsalmi and Balbaid, 2023; Elahdi and Alnahdi, 2022). In addition, limited proficiency in using technology has been reported (Alajami and Albatal, 2016; Algharbi and Alsayed, 2024). Deficits in social skills, including difficulty building relationships with colleagues and supervisors and participating in workplace activities, may further affect employment opportunities (AlDosari, 2016). These factors may contribute to employers' reluctance to hire individuals with intellectual disabilities (Alajami and Albatal, 2016; Amreen and Ghareeb, 2025).

### 3.2 What facilitators have been proposed to address the challenges of employing individuals with intellectual disability in Saudi Arabia, from the perspective of stakeholders?

The reviewed studies indicated several facilitators aimed at addressing employment challenges, as perceived by stakeholders (see Table 2). These facilitators can be grouped into two primary categories: collaborative partnerships and facilitative services and workplace relationships.

#### 3.2.1 Collaborative partnerships

The findings highlighted the need to establish a specialized government entity responsible for training individuals with intellectual disabilities and facilitating their access to employment opportunities (AlQahtani and AlDaaj, 2020; Amreen and Ghareeb, 2025). In addition, stakeholders emphasized the importance of an oversight body to monitor employment processes and ensure accountability for violations of their rights (AlQahtani and AlDaaj, 2020). The absence of such institutional structures has contributed to several challenges, including the lack of a centralized database of job seekers with intellectual disabilities (Alsharadqah and AlGhamdi, 2024) and limited follow-up on employment outcomes (Amreen and Ghareeb, 2025). Furthermore, relevant authorities need to provide material and informational support to employment sectors regarding necessary workplace accommodations, such as building modifications and flexible working hours, to facilitate access to employment (AlQahtani and AlDaaj, 2020).

Additionally, the findings emphasized the importance of establishing collaborative partnerships between schools and centers serving individuals with intellectual disabilities and local training and employment sectors. Such partnerships are essential for equipping individuals with the skills and practical experience required for successful labor market integration (Almalki et al., 2022; Amreen and Ghareeb, 2025; Baothman, 2021). The results also underscored the need for legislative action mandating structured training, such as requiring at least 1 year of vocational training during the final year of secondary education (Almalki et al., 2022). Addressing workplace discrimination and bias further requires the implementation of fair and transparent employment policies for individuals with disabilities (Abed et al., 2024). The absence of enforceable training requirements and the limited activation of supportive regulatory systems have weakened employer commitment to hiring individuals with intellectual disabilities (Abed et al., 2024; AlQahtani and AlDaaj, 2020).

#### 3.2.2 Accessibility services and relationships in the workplace

The findings emphasized the need to develop targeted training programs for employees without disabilities to foster inclusive workplace environments that value diversity and respect individuals regardless of ability (Abed et al., 2024). This need stems from limited awareness of how to effectively interact with employees with intellectual disabilities, low confidence in their work capabilities, and insufficient opportunities for skill development (AlQahtani and AlDaaj, 2020). In addition, colleagues without disabilities may avoid interaction, demonstrate low trust, marginalize employees with intellectual disabilities, and fail to assign meaningful responsibilities (AlDosari, 2016). These issues highlight the importance of awareness and educational initiatives for both employers and employees to challenge negative stereotypes and promote inclusive practices (Abed et al., 2024). Evidence also indicates that familiarity, frequent contact, and prior work experience with individuals with intellectual disabilities are associated with more positive attitudes toward their employment (Elahdi and Alnahdi, 2022).

The results further indicated that mismatches between job requirements and the abilities of individuals with intellectual disabilities limit employment opportunities (AlDosari and Muajini, 2019). In addition, the lack of accessible and supportive work environments presents significant barriers (Alsharadqah and AlGhamdi, 2024; Amreen and Ghareeb, 2025), including insufficient safety measures and the absence of secure working conditions (AlDosari and Muajini, 2019; Alsalmi and Balbaid, 2023). These challenges may contribute to parental concerns regarding workplace safety (AlQahtani and AlDaaj, 2020). Accordingly, the findings underscore the need to provide reasonable workplace accommodations and appropriate assistive technologies to support task performance and enhance employment outcomes for individuals with intellectual disabilities (Abed et al., 2024).

## 4 Discussion

This study aimed to identify the employment barriers faced by individuals with intellectual disabilities in Saudi Arabia and to propose facilitators to address them. A close analysis of the transcripts identified two central issues relevant to the research question: (1) interagency collaboration and access to training and (2) promoting inclusive work environments and corporate accountability.

### 4.1 Interagency collaboration and access to training

The findings identified several barriers associated with the limited vocational, social, and adaptive skills of individuals with intellectual disabilities. These barriers included weak communication and social interaction skills, difficulty adhering to expected workplace behaviors, managing daily tasks, building professional relationships, respecting colleagues' property, and following instructions accurately (Almalky and Alqahtani, 2022; Amreen and Ghareeb, 2025). Together, these limitations reduce employment opportunities and hinder labor market participation. Because these skills are essential for workplace success, they influence work readiness, relationships with colleagues, and long-term job retention.

These findings are consistent with global evidence indicating that individuals with intellectual disabilities often experience substantial deficits in vocational, social, and adaptive skills (Adams et al., 2019; Garrels et al., 2024; Meltzer et al., 2020). These deficits may reflect inadequate professional support in workplace settings (Adams et al., 2019; Elahdi and Alnahdi, 2022) and the limited role of educational systems in developing job-related competencies (Alzarie, 2024). Middle and high school curricula often emphasize academic achievement at the expense of life skills (Alfawzan and Almulhim, 2024; Alnahdi et al., 2024), with limited integration of vocational skills (Alsharbi and Alhuwaiti, 2022). In addition, limited opportunities for practical work experience outside school (Alharbi and Talafha, 2023) and insufficient emphasis on vocational preparation (Alnahdi, 2014; Carter et al., 2023) further reduce graduates' readiness for the labor market. Collectively, these factors contribute to negative employer perceptions of the employability of individuals with intellectual disabilities. This pattern is supported by the findings of Ali (2011) and Almughira (2024). Although these studies had some methodological limitations related to sample representation, their findings align with those of several high-quality studies included in this review (Abed et al., 2024; Alanazi, 2021; AlDosari, 2016; Alsharbi and Alhuwaiti, 2022; Amreen and Ghareeb, 2025), strengthening the consistency of the evidence and supporting the interpretation of the overall findings.

These issues reflect weaknesses in transition services and limited exposure to work-based learning (Elahdi and Alnahdi, 2022), which may stem from the absence of enforceable policies requiring collaboration between schools and community agencies during secondary education (Almalki et al., 2022). These findings underscore the critical role of legislation and transition planning teams in improving postsecondary outcomes and strengthening interagency collaboration. They also highlight the need for more structured transition planning and service delivery, which are essential for developing vocational skills (Elahdi and Alnahdi, 2022). Furthermore, integrating evidence-based transition practices into special education frameworks is necessary to improve employment outcomes (Kaabi and Alqudah, 2022). Work-based learning is recognized as an evidence-based practice for promoting successful post-graduation employment among individuals with disabilities (Wehman et al., 2015). It encompasses a range of practical experiences in authentic work settings that align with students' interests and preferences, including job shadowing, summer employment, and vocational training (Luecking et al., 2017). Participation in work-based learning enhances vocational and social skills while improving employment readiness (Riesen and Oertle, 2019).

Furthermore, interagency collaboration has consistently been identified as a key evidence-based predictor of successful post-school transition to employment for individuals with intellectual disabilities (Plotner et al., 2024; Test et al., 2009). Its effectiveness is supported by more than a decade of research demonstrating improved postsecondary outcomes for students with disabilities (Haber et al., 2016; Mazzotti et al., 2016; Noonan et al., 2008; Taylor et al., 2016). Individuals who received greater interagency support during high school were more than twice as likely to secure employment after graduation as those who received less support (Bullis et al., 1995). Interagency collaboration has long been recognized as a cornerstone of effective post-school transition planning (Meadows et al., 2014). Likewise, Kohler (1996) and Kohler et al. (2016) identified it as a core component of the Transition Programming Classification. Consistent with this evidence, the current study highlights the importance of establishing coordinated partnerships among schools, centers serving individuals with intellectual disabilities, and employment agencies to strengthen vocational skill development and facilitate successful labor market integration (Almalki et al., 2022; Amreen and Ghareeb, 2025; Baothman, 2021).

In addition, legislative frameworks support these practices. The U.S. Individuals with Disabilities Education Act (IDEA) emphasizes the importance of involving non-school stakeholders in Individual Transition Plan (ITP) meetings (Plotner et al., 2024). Such collaboration helps students with intellectual disabilities identify appropriate training opportunities within authentic transition settings during secondary education. These findings also align with Saudi Arabia's Vision 2030, which seeks to expand access to appropriate education and employment opportunities for individuals with disabilities by equipping them with the skills needed for successful workforce participation (Saudi Arabia's Vision 2030, 2016). Similarly, the 2023 amendment to the Individuals with Disabilities Law reinforces this commitment. Article 10 emphasizes the development and implementation of employment, vocational, and technical training programs, which may further encourage employers to recruit and retain individuals with disabilities (System for Rights of Persons with Disabilities, 2023).

Similarly, Article 2 of the Saudi System for the Care of Persons with Disabilities emphasizes the importance of enhancing workplace performance through training (Disabled Care System, 2000). In practice, the Ministry of Human Resources and Social Development provides a range of vocational training opportunities through specialized vocational and employment centers tailored to the abilities and needs of individuals with disabilities. The Human Resources Development Fund (HRDF) further supports participation in training, rehabilitation, and employment initiatives through programs such as Tawafuq and the national e-training platform Doroob, which has been adapted to meet the needs of individuals with disabilities. Collectively, these initiatives aim to enhance job performance, promote self-reliance, and support the integration of individuals with disabilities as productive members of society.

Although policies addressing the multiple barriers to employment are essential, they are insufficient on their own. Individuals with intellectual disabilities continue to experience high rates of unemployment, underscoring the need for early work-based learning opportunities during the transition to adulthood (Riesen and Oertle, 2019). Secondary schools should prioritize early and continuous field-based training by organizing regular visits to workplaces, including institutions, factories, and companies, to increase familiarity with authentic work environments (Alfarran, 2021). Such experiences help individuals acquire vocational skills and develop a sense of pride (Riesen and Oertle, 2019). In addition, Elahdi and Alnahdi (2022) emphasized the importance of providing training programs for families to strengthen their capacity to support vocational preparation, particularly in light of the limited family involvement identified in the findings.

While these efforts reflect a clear commitment to promoting employment and social inclusion, several challenges remain, particularly regarding the availability and accessibility of services. The current findings indicate that individuals with intellectual disabilities are often absent from employment platforms and workshops, which may reflect transportation barriers and limited access to the training and support services necessary for successful employment (Adams et al., 2019; Bialik and Mhiri, 2022). Although the HRDF Wusool Transportation Support Initiative seeks to reduce transportation costs for employees with disabilities by facilitating access to workplaces through partnerships with licensed ride-hailing services (Human Resources Development Fund, 2025), more coordinated efforts are needed to increase awareness of the initiative and expand its reach.

### 4.2 Promoting inclusive work environments and corporate accountability

The findings highlighted the need to develop training programs for employees without disabilities to promote inclusive workplace environments that respect diversity and value individual differences (Abed et al., 2024). This need reflects limited understanding of inclusive employment approaches (Fajardo-Castro et al., 2024; Jacob et al., 2023; Morris et al., 2024) and insufficient awareness among some employers of how to effectively support employees with intellectual disabilities (AlQahtani and AlDaaj, 2020). These gaps may undermine confidence in the capabilities of employees with intellectual disabilities and discourage meaningful workplace interaction and engagement (AlDosari, 2016; AlQahtani and AlDaaj, 2020). Consequently, they constitute significant barriers to employment and workplace integration for individuals with intellectual disabilities (Fajardo-Castro et al., 2024; Jacob et al., 2023; Morris et al., 2024). These findings are consistent with evidence from other contexts emphasizing the importance of workplace education and awareness in addressing misconceptions and fostering inclusive practices (Bialik and Mhiri, 2022; Jacob et al., 2023; Morris et al., 2024).

Accordingly, educating both employers and employees is essential to challenge negative stereotypes, improve workplace interactions, and foster more supportive and inclusive work environments (Abed et al., 2024). The inclusion of individuals with disabilities, particularly those with intellectual disabilities, contributes to a more supportive and adaptable workplace culture while enhancing employee wellbeing through diversified tasks and collaborative work environments (Joyce et al., 2024). It also strengthens organizational culture, promotes social responsibility, and increases colleagues' understanding of and empathy toward individuals with intellectual disabilities (Lindsay et al., 2018; Riesen and Oertle, 2019), reinforcing that employing individuals with intellectual disabilities is not only a socially just practice but also a source of meaningful organizational value.

In this context, the Saudi Arabian government has taken concrete steps to promote the employment of individuals with disabilities. A key provision is Article 28 of the Labor Law, which requires employers with 25 or more employees, where job roles permit, to ensure that at least 4% of their workforce comprises individuals with disabilities while providing appropriate workplace accommodations (Board of Experts at the Council of Ministers, 2005). In response to employer concerns about the costs of inclusion, the government has introduced financial support mechanisms to facilitate training and employment (Ministry of Human Resources and Social Development, n.d.). In addition, the HRDF supports employers through the Employment Support program by subsidizing wages for Saudi employees with disabilities, including an additional 10% contribution alongside broader wage subsidies ranging from 30% to 50% (AlQahtani and AlDaaj, 2020; Kaabi and Alqudah, 2022). The Ministry of Human Resources and Social Development further promotes inclusive employment through initiatives that help organizations adapt their work environments. One such initiative is the Mowaamah certification program, implemented under the Tawafuq initiative in collaboration with the HADAF. This voluntary certification recognizes organizations committed to providing accessible environments and inclusive practices that support individuals with disabilities (Mowaamah, 2025).

However, the current findings indicate that unsuitable working conditions and inaccessible physical environments remain significant barriers to the employment of individuals with intellectual disabilities (Alsharadqah and AlGhamdi, 2024; Amreen and Ghareeb, 2025). These findings are consistent with evidence from other contexts demonstrating persistent gaps in the provision of appropriate facilities and workplace accommodations (Bialik and Mhiri, 2022; Carter et al., 2023; Fajardo-Castro et al., 2024; Morris et al., 2024). Together, they underscore the need for stronger governmental oversight to ensure effective implementation of existing legislation and hold organizations accountable for non-compliance. In addition, the Ministry of Human Resources and Social Development should strengthen its efforts to create supportive and motivating work environments by introducing and enforcing policies that promote the employment of individuals with intellectual disabilities across the public and private sectors (Elahdi and Alnahdi, 2022).

## 5 Implications and future studies

### 5.1 Evidence based recommendations

In recent years, Saudi Arabia has made significant progress in addressing the challenges faced by individuals with intellectual disabilities. Despite these advances, the findings underscore the need to raise societal awareness of the labor rights of individuals with intellectual disabilities and promote their inclusion in the labor market. The findings also revealed limited employer preparedness and insufficient knowledge of intellectual disability, highlighting the need to equip employers with the knowledge and skills required to foster workplace diversity, recognize the contributions of individuals with intellectual disabilities to organizational performance, and appreciate how their inclusion promotes empathy, strengthens understanding of diversity, and enhances acceptance within inclusive work environments. Adapting working conditions and ensuring accessible work environments remain essential for sustaining employment. In addition, the findings identified persistent challenges related to workplace communication, social interaction, and adaptability among individuals with intellectual disabilities.

### 5.2 Broader implications and interpretive recommendations

Accordingly, this study recommends revising secondary education curricula to strengthen social, vocational, and adaptive skills in line with labor market demands and improve employment opportunities. The findings also revealed gaps in transition planning and weak interagency collaboration, underscoring the need to strengthen legislation and policies that facilitate a successful transition from secondary education to employment, particularly those supporting vocational training and interagency collaboration. Such measures would enable individuals with intellectual disabilities to access meaningful employment opportunities and participate more fully in society. Finally, the study recommends conducting a 10-year longitudinal study to evaluate the effectiveness and sustainability of employment services provided to individuals with intellectual disabilities after graduation.

## 6 Limitations

This systematic review has several limitations that should be considered when interpreting the findings. First, the review is geographically limited to studies conducted in Saudi Arabia, and several included studies relied on relatively small sample sizes, which may restrict the generalizability of the findings. Second, only studies published in Arabic or English were included, excluding unpublished research and studies published in other languages. Consequently, the available evidence on the employment of individuals with intellectual disabilities may be broader than represented in this review. Third, although the review identified clear employment challenges, relatively few studies examined potential solutions, with only 11 addressing strategies to overcome these barriers. Fourth, although the review included studies reflecting the perspectives of individuals with intellectual disabilities, their representation in the literature remained limited compared with that of employers, teachers, and parents. This underscores the need for further research that gives greater prominence to their perspectives. Fifth, the inclusion criteria were limited to studies that explicitly identified their samples as individuals with intellectual disabilities. Consequently, broader research involving individuals with disabilities was excluded. Future research should integrate this broader literature while examining subgroup-specific findings to provide a more comprehensive understanding of employment experiences.

Additionally, the review has temporal limitations because several included studies were conducted during earlier periods, particularly around 2011. This may reduce the relevance of some findings to the current context, especially in light of recent legislative and societal developments in Saudi Arabia supporting the employment of individuals with intellectual disabilities. Despite these limitations, the review adhered to the PRISMA guidelines (Liberati et al., 2009), and study selection followed a structured, systematic methodology to address the research questions with rigor and consistency.

## 7 Conclusion

This review of published research on the employment of individuals with intellectual disabilities in Saudi Arabia identified numerous barriers alongside a range of facilitators to address these challenges. It incorporated the perspectives of employers, human resources managers, teachers, supervisors, trainers, rehabilitation staff, and parents, as well as five studies that captured the voices of individuals with intellectual disabilities, whose contributions provided valuable insights into their employment experiences. The findings underscore the urgent need to strengthen vocational training and transition services to better prepare individuals for labor market participation while fostering inclusive workplace environments that value diversity. The review also highlights the importance of enforcing existing legislation and establishing effective institutional accountability mechanisms to address the long-term personal, economic, and societal consequences of employment barriers. Despite these challenges, there is considerable potential for progress, supported by growing recognition that individuals with intellectual disabilities make valuable contributions to the workforce and national economic development, consistent with Saudi Arabia's Vision 2030. The findings are expected to inform policymakers, researchers, rehabilitation and training providers, and individuals with intellectual disabilities, thereby supporting more effective strategies to promote employment inclusion.

## Data Availability

The original contributions presented in the study are included in the article/supplementary material, further inquiries can be directed to the corresponding author.
